# The Participation of People with Disabilities in the Workplace Across the Employment Cycle: Employer Concerns and Research Evidence

**DOI:** 10.1007/s10869-018-9602-5

**Published:** 2019-01-22

**Authors:** Silvia Bonaccio, Catherine E. Connelly, Ian R. Gellatly, Arif Jetha, Kathleen A. Martin Ginis

**Affiliations:** 1 Telfer School of Management, University of Ottawa, 55 Laurier Avenue East, Ottawa, ON K1N 6N5 Canada; 2 DeGroote School of Business, McMaster University, Hamilton, Canada; 3 Alberta School of Business, University of Alberta, Edmonton, Canada; 4 Institute for Work & Health, Toronto, Canada; 5 Dalla Lana School of Public Health, University of Toronto, Toronto, Canada; 6 School of Health and Exercise Sciences, University of British Columbia, Vancouver, Canada

**Keywords:** Disabilities, Diversity, Employment discrimination, Employment cycle

## Abstract

Despite legislation on diversity in the workplace, people with disabilities still do not experience the same access to work opportunities as do their counterparts without disabilities. Many employers have been shown to harbor sincere yet ill-founded views about the work-related abilities of people with disabilities; these negative views are often a result of interrelated concerns that permeate the entire employment cycle. In this paper, we provide evidence-based responses to 11 specific concerns that employers have about people with disabilities, from pre-employment and entry experiences to the final dissolution of the employment relationship. At each stage of the employment cycle, we summarize and evaluate the relevant empirical evidence and provide recommendations for organizations committed to creating more effective, equitable, and inclusive workplaces for all individuals. We also suggest avenues for future research.

For many people with disabilities, finding and sustaining work is a challenge. Indeed, it has been estimated that in the United States (US), only one in three (34.9%) individuals with disabilities are employed compared to 76% of their counterparts without disabilities, and this disparity appears to be increasing over time (Houtenville & Ruiz, 2012; Kraus, 2017; Lauer & Houtenville, 2017). Similar employment gaps have been observed in other industrialized countries. For instance, the employment rate among working-age Canadians living with a disability is 49%, while it is 79% for those without a disability (Turcotte, 2014), and in the European Union, these figures are 47.3 and 66.9%, respectively (Eurostat, 2017). While the World Health Organization (WHO, 2011) shows that employment rates vary across countries, “the bottom line is that, all over the world, a person with a disability is less likely to be employed than a person without a disability, often much less so” (Heymann, Stein, & de Elvira Moreno, 2014, p. 4). Even when employed, workers with disabilities are more likely than their counterparts without disabilities to report underemployment, involuntary part-time or contingent employment, and lower than average salaries (Brault, 2012; Konrad, Moore, Ng, Doherty, & Breward, 2013; see also Baldridge, Beatty, Konrad, & Moore, 2016). Notwithstanding legislation specifically targeted at promoting and protecting the rights of people with disabilities (e.g., Americans with Disabilities Act [1990] of 1991), the employment participation of people with disabilities is still lagging when compared to their able-bodied, and comparably educated, counterparts (WHO, 2011; see also Colella & Bruyère, 2011; Kruse & Schur, 2003).

A primary reason for the lower participation rates and underemployment of individuals with disabilities is that employers often harbor pessimistic views about the work-related abilities of these individuals. We note that these pessimistic views have been well-documented in the literature (e.g., Gold, Oire, Fabian, & Wewiorksi, 2012; Hernandez et al., 2008; Kaye, Jans, & Jones, 2011; Lengnick-Hall, Gaunt, & Kulkarni, 2008; see also white papers by Domzal, Houtenville, & Sharma, 2008; Gaunt & Lengnick-Hall, 2014). What is missing is an in-depth analysis of *where* in the employment relationship employers’ pessimistic views appear, and whether these concerns are supported by empirical evidence.

In this article, we provide an organizing framework to understand where employers’ views are likely to have the greatest implications for persons with disabilities. We do so by mapping employer concerns onto the management practices associated with each stage of the employment cycle, which is described in the next section. For each employment cycle stage, we summarize and evaluate the relevant empirical evidence and provide recommendations for organizations committed to creating more effective, equitable, and inclusive workplaces for all.

To locate source material for our analyses, we conducted cited reference searches of key empirical papers documenting employers’ pessimistic views (Kaye et al., 2011; Lengnick-Hall et al., 2008) and a classic review paper (Stone & Colella, 1996) pertaining to workers with disabilities. We also reviewed more recent handbook chapters and review articles (e.g., Baldridge et al., 2016; Colella & Bruyère, 2011; Santuzzi & Waltz, 2016) to locate relevant primary research about each concern. Finally, given that research on workers with disabilities spans several fields, we used several databases: PsycINFO, Scopus, EBSCO, PubMed, and Medline, as well as Google Scholar, using keywords related to disability topics (i.e., accommodation, disability, participation barrier) along with keywords related to each employment cycle stage, in turn, to locate additional primary research. We integrated the current literature in human resources, management, and industrial/organizational psychology with research in other fields (e.g., rehabilitation sciences, public health).

## The Employment Cycle

**Fig. 1:**
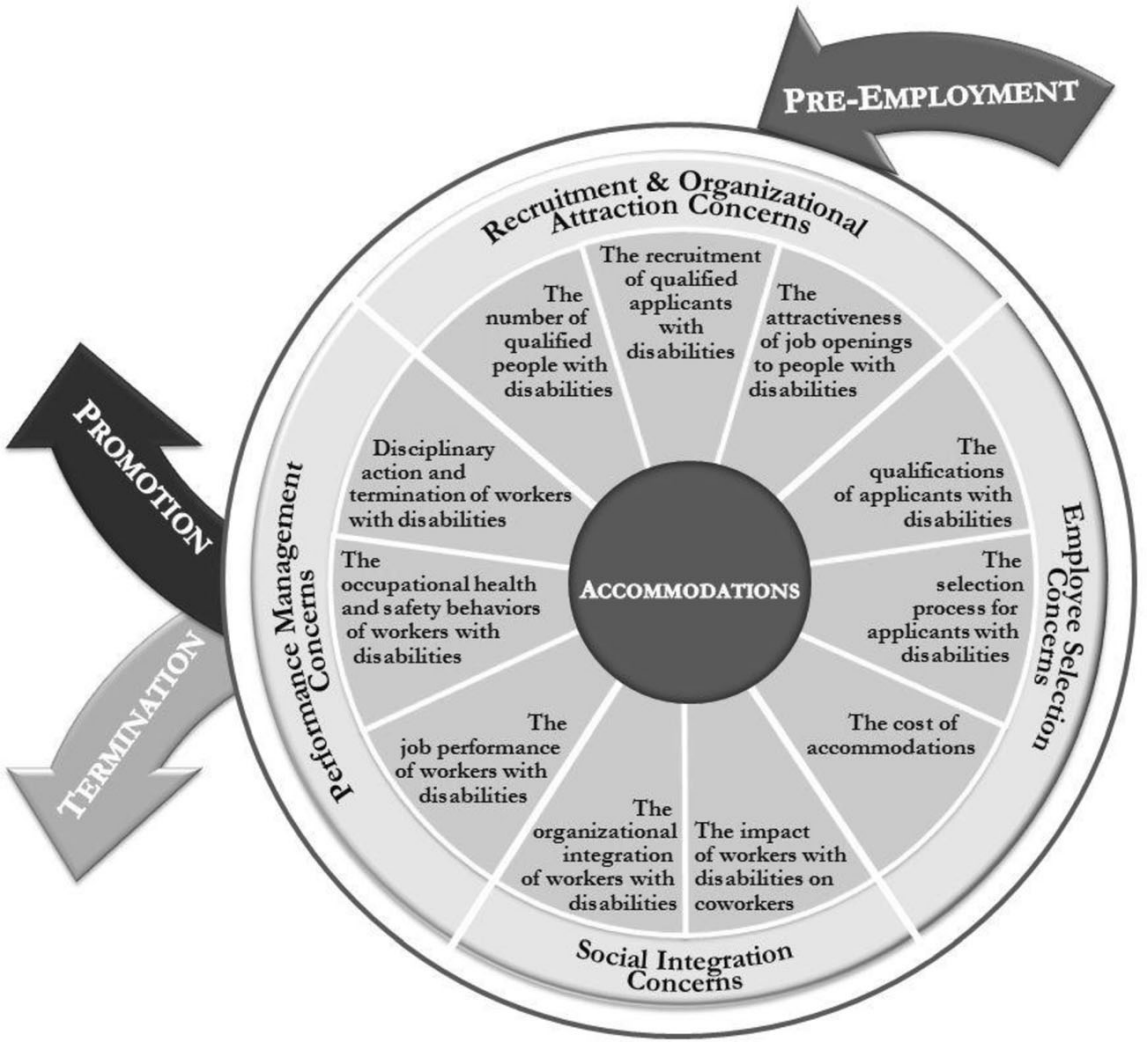
The employment cycle and employers’ concerns about people with disabilities

We have organized managers’ concerns about the suitability of people with disabilities by following the typical course of the employment relationship (e.g., recruitment, selection, social integration, performance management). Figure 1 illustrates the employment cycle, along with the relevant concerns that managers may have at each stage of the process. We assume that the employment relationship begins when both parties first become aware of each other’s existence, reflected in the goals of anticipatory socialization (from the prospective member’s perspective) and active recruitment (from the employer’s perspective). At this stage, the labor supply and the ease of reaching appropriate applicant pools may be of concern. Indeed, managers may wonder whether people with disabilities are even available, and, if so, whether recruiting from this labor pool is complicated. Managers may further ask whether people with disabilities would be interested in their job openings. From a selection perspective, managers may question whether applicants with disabilities would actually have the right qualifications. Managers may also be concerned that they would have to change their recruitment approach if they encounter an applicant with a disability. Underlying most HR processes, from encounter to separation, is the topic of accommodations, and we address this concern at the moment in which accommodations may first be discussed: during the selection stage. Once selected, the employee and employer move into the actual employment relationship, during which social integration and performance management are key elements. Here, managers may be unclear about the impact the newly hired employee may have on existing employees. Furthermore, managers may express concerns about workers with disabilities’ performance and safety behaviors. If performance problems do occur, managers may be unsure as to how to address them, or, in the event that they persist, how to terminate the employment relationship.

## Recruitment and Organizational Attraction

### Concern 1: the Number of Qualified People with Disabilities

Past research has found that managers report that they “rarely see” workers with disabilities in their applicant pools (Kaye et al., 2011, p. 528). We contend that managers may be underestimating how many workers with disabilities apply for their job openings. This contention is better understood when considering the prevalence of people with disabilities within the labor pool. For example, between 10 and 16% of working-age Americans report having a disability (Brault, 2012; Kraus, 2017; Lauer & Houtenville, 2017; Stevens et al., 2016). These figures are not unlike those in other Western countries.^1^ For example, in Canada, about 11% of the working-age population reports living with a disability (Turcotte, 2014), 16% do so in the UK (Department for Work & Pensions, 2014), and 15% do so in the Netherlands and in Sweden (Statistics Netherlands, 2010; Statistics Sweden, 2017). While these figures represent all types of disabilities, physical disabilities are the most prominent type of disability among people of working age (Arim, 2015; Australian Bureau of Statistics, 2016; Kraus, 2017; Statistics Sweden, 2017).

Given the prevalence of disabilities, and irrespective of the type of disability, it is quite likely that applicant pools contain more people with disabilities than may be obvious to managers. There are at least three interrelated explanations why hiring managers may underestimate how many workers with disabilities are in their applicant pools.^2^ First, many disabilities are not easily discernable and are effectively “invisible” to all but the affected party. Included in invisible disabilities are “a wide range of physical and psychological conditions that often have no visible manifestation or have visible features that are not clearly connected to a disability” (Santuzzi, Waltz, Finkelstein, & Rupp, 2014, p. 204), such as diabetes, arthritis, and depression. In many instances, workers with invisible disabilities might be able to conceal their disabilities quite readily from interviewers, coworkers, and supervisors, as in the case of a person with hearing loss who relies on lip reading (e.g., Jans, Kaye, & Jones, 2012) and employees living with mental illnesses (Elraz, 2018). Many disabilities are also episodic such that individuals experience fluctuations in symptom severity. For example, individuals may report minor to severe fluctuations in well-being on daily (e.g., feeling worse at the end of the day), weekly (e.g., feeling worse as the week progresses), and monthly (e.g., feeling better as one recovers from treatments) cycles. Santuzzi et al. (2014) argue that estimates of the proportion of the workforce that has a disability are actually *underestimates*, in part because of invisible disabilities. If the prevalence of people with disabilities in the workforce recorded in systematic government surveys is underestimated, it is inevitable that individual managers will also underestimate the number of workers with disabilities in their own applicant pools.

Second, the issue of disclosure is related to the discussion of invisible disabilities, and it may help to explain why managers may be unaware of the actual number of workers with disabilities in their applicant pools. In some cases, people may choose to conceal their disabilities because they fear negative repercussions on their careers should they disclose them (Barclay & Markel, 2007; Jans et al., 2012; Santuzzi et al., 2014; see also Ragins, 2008). In other cases, they do not want to feel different from their peers (Jetha, Bowring, Tucker, et al., 2018). Because of these concerns, applicants with disabilities may forego disclosure unless an accommodation is necessary, although they may also forego disclosure even if it means withholding accommodation requests (Gignac, Cao, & McAlpine, 2015; Jans et al., 2012; Santuzzi et al., 2014). Employees’ concerns surrounding disclosure are valid; there is evidence that some managers discriminate against individuals with disabilities (Kaye et al., 2011) or make different employment decisions based on disability status (Premeaux, 2001; see also Hayes & Macan, 1997). However, managers may also react negatively to a late disclosure (Gold et al., 2012; Jans et al., 2012), even when “late” simply means noting one’s disability at the end of an employment interview instead of the beginning (Hebl & Skorinko, 2005). The decision whether to disclose, when to disclose, and to whom to disclose is deeply personal (Jans et al., 2012; Von Schrader, Malzer, & Bruyère, 2014), and it is more difficult if one’s condition is stigmatized (e.g., HIV/AIDS; Ragins, 2008). Thus, people with disabilities are often advised against disclosing in the early stages of the employment relationship (e.g., the interview) and to either disclose after an employment offer or not at all until accommodations are needed (e.g., Von Schrader et al., 2014). Finally, managers may underestimate the number of people with disabilities in their applicant pool because their recruitment practices inadvertently deter people with disabilities from applying in the first place (Bruyère, Erikson, & VanLooy, 2005). We turn to this point in the next section.

#### Practical Implications and Research Directions

In summary, managers may underestimate the number of people with disabilities in applicant pools. Many government organizations and community organizations have developed resources to counteract the lack of awareness that managers (and coworkers) display toward workers with disabilities. These resources are essential because both employers (Kaye et al., 2011) and employees with disabilities (Wilson-Kovacs, Ryan, Haslam, & Rabinovich, 2008) report employers’ lack of disability-related knowledge, which has implications for their behavior. These resources are intended to assist employers to increase their knowledge of disabilities, and improve their competence in interacting with people with disabilities in a work context. For example, the Job Accommodation Network (JAN), a free consulting service provided by the Office of Disability Employment Policy of the US Department of Labor, provides information and offers a webcast on language and etiquette. Another service provided by this office, the Employer Assistance and Resource Network on Disability Inclusion (EARN), also gives advice on language and especially the importance of inclusive and people-first language, something also noted by the Australian Network on Disability (AND). Both JAN and AND provide facts about disabilities. For example, AND provides a concise overview of disability types, and JAN offers information on more than 100 disabilities and functional limitations and suggested accommodations. For all 11 concerns, readers can find descriptions and hyperlinks for the resources mentioned in the “Practical Implications and Research Directions” sections along with additional resources in Table 1.

**Table 1:** Online resources

Resource	Web Address	Description
**Concern 1:*The number of qualified people with disabilities***		
Australian Network on Disability (AND)	www.and.org.au	Organization for inclusion of people with disabilities in business
	www.and.org.au/pages/inclusive-language.html	Factsheet on inclusive language
	www.and.org.au/pages/what-is-a-disability.html	Factsheet on defining disability
Disability Statistics	www.disabilitystatistics.org	Multi-source national and state level data for multiple variables
Employer Assistance and Resource Network on Disability Inclusion (EARN)	www.askearn.org	Department of Labor resources to help employers make businesses disability inclusive
	www.askearn.org/topics/retention-advancement/disability-etiquette	Advice about disability etiquette
	www.askearn.org/topics/retention-advancement/disability-etiquette/people-first-language	Resource about people-first language
	www.askearn.org/wp-content/uploads/2016/07/EARN-Self-ID-Fact-Sheet.pdf	Factsheet for employers about encouraging employees with disabilities to self-identify
	www.askearn.org/wp-content/uploads/docs/do_ask_do_tell.pdf	Report for employers about encouraging employees with disabilities to self-identify
	www.askearn.org/training-center/webinars/do-ask-do-tell-tapping-the-power-of-disability-diversity-encouraging-self-identification	Webinar for employers about encouraging employees with disabilities to self-identify
	www.askearn.org/training-center/webinars/disclosing-disability-what-you-need-to-know	Webinar for employees about disclosure
Job Accom-modation Network (JAN)	https://askjan.org	Department of Labor service for guidance about accommodations and disability employment
	https://jan.adobeconnect.com/aware/	*Disability Awareness to Increase Your Comfort,* *Confidence and Competence* webcast
	https://askjan.org/links/atoz.htm	Information listed by disability, topic, or functional limitation and suggested accommodations.
Office of Disability Employment Policy	www.dol.gov/odep/topics/disability.htm	Department of Labor disability resources list
**Concern 2:*The recruitment of qualified applicants with disabilities***		
Canadian Council on Rehabilitation and Work	www.workink.com	Job postings by equity employers
EARN	www.askearn.org/training-center/webinars/ensuring-virtual-doors-open-key-component-disability-inclusion	Webinar on e-recruitment
	www.askearn.org/inclusion-work	*Inclusion@Work* series of modules with steps for building disability-inclusive businesses
	www.askearn.org/training-center/webinars/building-the-pipeline-successful-strategies-for-recruiting-hiring-people-with-disabilities	Webinar on recruitment and hiring
	www.askearn.org/StepsToSuccess/small-businesses	Toolkit for small businesses about effective employment practices
	www.askearn.org/topics/recruitment-hiring/finding-candidates-with-disabilities	List of resources for finding candidates with disabilities
Government of Canada	www.publications.gc.ca/collections/collection_2013/rhdcc-hrsdc/HS64-21-2013-eng.pdf (Fredeen et al., 2013)	Report from the Panel on Labor Market Opportunities for Persons with Disabilities
	www.publications.gc.ca/collections/collection_2011/cfp-psc/SC3-156-2011-eng.pdf	Literature review on recruiting persons with disabilities; great list of resources in bibliography
Kulkarni & Kote, 2014	https://doi.org/10.1007/s10672-013-9216-z	Descriptions of services offered by placement agencies
National Organization on Disability	www.nod.org/wp-content/uploads/02_best_practices_for_recruiting_students_with_disabilities.pdf	Guide to recruiting students with disabilities
	www.nod.org/wp-content/uploads/04_employer_driven_initiatives.pdf (Katz, O’Connell, & Nicholas, 2012)	*Strategies to Support Employer-Driven Initiatives to Recruit and Retain Employees with Disabilities*
**Concern 3:*The attractiveness of job openings to people with disabilities***		
EARN	www.askearn.org/inclusion-work/inclusive-business-culture	Module on building an inclusive work culture
	www.askearn.org/training-center/webinars/lead-the-way-building-an-inclusive-business-culture	Webinar on building an inclusive work culture
	www.askearn.org/topics/recruitment-hiring/expressing-a-commitment-to-disability-inclusion	Advice about how to express a commitment to an inclusive workplace
ADA National Network	https://adata.org/factsheet/section-503	Fact sheet on Section 503 of the Rehabilitation Act
**Concern 4:*The qualifications of applicants with disabilities***		
After College	www.employer.aftercollege.com/resources-hiring-students-disabilities/	Resource for employers about hiring students and alumni with disabilities
EARN (Heidkamp & Hilliard)	www.askearn.org/wp-content/uploads/2018/03/A-Review-of-Community-College-Employer-Partnerships-and-Initiatives-Final.pdf	Review of employer-educational institution partnership types and list of partnerships
Humber	www.careers.humber.ca/disabilities-info-employer.php	College site for employers about hiring students and alumni with disabilities
University of Guelph	www.recruitguelph.ca/cecs/employers-institutions/hiring-students-disabilities	University site for employers about hiring students and alumni with disabilities
University of Washington	https://careers.uw.edu/employers/candidates-with-disabilities	University site for employers about hiring students and alumni with disabilities
Workforce Recruitment Program	https://wrp.gov	Recruitment and referral program to connect employers with students and alumni with disabilities
**Concern 5:*The selection process for applicants with disabilities***		
EARN	www.askearn.org/topics/recruitment-hiring/interviews	Advice about interviewing candidates with disabilities
Hire for Talent	https://hirefortalent.ca/main/toolkit/interviews	Toolkit for employers about interviewing candidates with disabilities
AND	www.and.org.au/pages/interviewing-people-with-disability.html	Advice about interviewing candidates with disabilities
**Concern 6:*The cost of accommodations***		
Australian Government	www.jobaccess.gov.au/employers/available-support/191	Financial support for hiring people with disabilities
Canadian Human Rights Commission (CHRC)	www.chrc-ccdp.gc.ca/eng/content/webinars	Series of webinars on the ‘duty to accommodate’
EARN	www.askearn.org/topics/laws-regulations/employer_financial_incentives	List of employer financial incentives, federally and state by state
	www.askearn.org/resources/employer-success-stories	Business success stories
*Equal Employment Opportunity Commission*	www.eeoc.gov/facts/accommodation.html	Guide for small businesses about ‘reasonable accommodations’
Gaunt & Lengnick-Hall, 2014	www.cprf.org/studies/overcoming-misperceptions-about-hiring-people-with-disabilities/	Tax incentives for hiring people with disabilities described in appendix
Government of Canada	www.canada.ca/en/services/business/hire/wagesubsidiesotherassistanceprograms.html	List of employer incentives, some of which prioritize hiring people with disabilities
	www.publications.gc.ca/collections/collection_2011/cfp-psc/SC3-156-2011-eng.pdf	Accommodation resources in appendix
JAN	https://askjan.org	Department of Labor service for guidance about accommodations and disability employment
	https://askjan.org/links/atoz.htm	Information about accommodations listed by disability or functional limitations
Office of Disability Employment Policy	www.dol.gov/odep/topics/Accommodations.htm	Resources on ‘reasonable accommodations’
US Chamber of Commerce	www.uschamber.com/sites/default/files/legacy/reports/DisabilityInclusionreport.pdf	Report about successful disability inclusion strategies
**Concern 7:*The impact of workers with disabilities on coworkers***		
CHRC	www.chrc-ccdp.gc.ca/eng/content/webinars	Webinar on inclusive workplaces
EARN	www.askearn.org/state-vocational-rehabilitation-agencies	List of vocational rehabilitation agencies by state, many of which offer services to employers
Hire for Talent	https://hirefortalent.ca/main/services	List of organizations providing services to employers by province
	https://hirefortalent.ca/main/toolkit/inclusive-workplaces/174-what-is-an-inclusive-workplace-policy	Tool for creating an inclusive workplace policy
Workplace Initiative	https://workplaceinitiative.org/disability-plan	Guide to choosing a service provider
**Concern 8:*The organizational integration of workers with disabilities***		
Employer Assistance and Resource Network on Disability Inclusion (EARN)	http://www.askearn.org/wp-content/uploads/docs/askearn_employeeresourcegroup_factsheet.pdf	Factsheet about employee resource groups
	www.askearn.org/wp-content/uploads/docs/erg_toolkit.pdf	Toolkit for creating employee resource groups
	www.askearn.org/inclusion-work/inclusive-business-culture	Module on building an inclusive work culture
	http://www.askearn.org/topics/creating-an-accessible-and-welcoming-workplace/attitudinal-awareness	Advice on breaking down attitudinal barriers
National Organization on Disability	www.nod.org/services/tracker/	*Disability Employment Tracker*: Free assessment tool for benchmarking disability inclusion
	http://www.nod.org/wp-content/uploads/2019-NOD-Disability-Employment-Tracker-FAQ-Document.pdf	Guide about *Disability Employment Tracker*
**Concern 9:*The job performance of workers with disabilities***		
EmployAbi-lities	https://employabilities.ab.ca/tim-hortons-hires-people-with-disabilities	Story of a restaurant franchisee known for his inclusive hiring practices
Ontario Disability Employment Network	www.odenetwork.com/businesses/why-hire	Guide for employers about the benefits of hiring people with disabilities
	www.youtu.be/giv7cMhGxlI	Video for employers about hiring people with disabilities, with employer testimonials
Workplace Initiative	https://Workplaceinitiative.org/case-studies	Profiles of employer disability-inclusion initiatives
**Concern 10:*The occupational health and safety behaviors of workers with disabilities***		
AND	www.and.org.au/pages/evacuation-procedures.html	Guide to inclusive evacuation procedures
Health & Safety Ontario (Micheelsen & Williams)	http://www.wsps.ca/HSO/media/HSO/NetworkMag/NetworkMag2/Article3a.html	Guide to creating individualized workplace emergency response information
**Concern 11:*Disciplinary action and termination of workers with disabilities***		
ADA National Network	https://adata.org/ada-training	Overview of available ADA training
	https://adata.org/technical-assistance	Contact information for ADA guidance
Australian Human Rights Commission	www.humanrights.gov.au/employers/join-network	*Business and Human Rights Network* for employers, as well as academics and others
CHRC	www.chrc-ccdp.gc.ca/eng/content/webinars	Webinars on inclusive workplaces, including on the complaint process
EARN	www.askearn.org/topics/laws-regulations	List of disability-related laws and regulations sites
Government of Canada	www.canada.ca/en/treasury-board-secretariat/services/values-ethics/diversity-equity/duty-accommodate-general-process-managers.html	Guide for managers about the ‘duty to accommodate’
Health and Safety Executive	www.hse.gov.uk/disability/law.htm	Guide to disability-related employment laws
SenseAbility	https://senseability.ca/about-us	Business network for disability-inclusive employers
Disability:IN	https://disabilityin.org	Group representing disability-inclusive businesses and businesses owned by people with disabilities

As part of this awareness building, employers must appreciate the variable nature of disabilities. Many disabilities and their presentation (e.g., arthritis, diabetes) will change over a person’s lifespan and career, which means that the interaction between disability and work factors varies considerably (Jetha, Bowring, Tucker, et al., 2018). Furthermore, the likelihood of developing or acquiring a disability increases with age (Kraus, 2017; Lauer & Houtenville, 2017), so disability likelihood is positively related to career stage (Jetha, Besen, & Smith, 2016). Thus, an employee with no disabilities at the time of hire can develop a disability gradually (e.g., progressive hearing loss) or suddenly (e.g., impairments caused by work or nonwork trauma). Finally, the symptoms of some disabilities are episodic. Episodic disabilities may be particularly difficult for managers to recognize because their impact on workers’ activities may fluctuate significantly.

The invisible or variable nature of many disabilities often places the onus of disclosure on employees. Disclosure is a personal decision that can be difficult, and employers are often ill-prepared to participate in a disclosure discussion that will result in positive long-term outcomes for both parties involved. Because of this, some resources have been developed to support disclosure discussions, such as a factsheet and a report documenting organizational best practices on disclosure, both developed by EARN.

From a research perspective, more empirical work is needed to expand our understanding of when, how, and why employees disclose invisible disabilities. Studies of the disclosure of other invisible stigmatized identities have been informative (e.g., Chaudoir & Fisher, 2010; Ragins, 2008). Particularly useful has been the work that has focused more specifically on the disclosure of disabilities (e.g., Beatty & Kirby, 2006; Clair, Beatty, & MacLean, 2005). Still, more research is needed to better understand how and when individuals disclose disabilities over time. For instance, the level of interpersonal and organizational trust might be found to play a key role in determining when people feel comfortable enough to disclose sensitive information about themselves. Furthermore, the disclosure of disabilities is potentially different than the disclosure of other identities (e.g., sexual orientation: King, Reilly, & Hebl, 2008) given managers’ specific concerns surrounding the performance of workers with disabilities, a point we discuss in a later section. Thus, understanding with whom disclosure occurs (e.g., direct manager, colleagues, HR department personnel) and the discourse strategies employed by workers in each of these discussions are important. This work is crucial because there are differential outcomes for acknowledging or disclosing disabilities depending on the strategy employed or the type of disability discussed (Lyons et al., 2016; Lyons, Volpone, Wessel, & Alonso, 2017). As a result, managers at all levels play an important role in ensuring an organizational culture/climate that makes disclosure and acknowledgement discussions safe and constructive.

### Concern 2: the Recruitment of Qualified Applicants with Disabilities

Managers, owners, and HR personnel who are tasked with selecting new employees understandably want to identify the best candidate for each job opening. One way to address this is by recruiting broadly, sourcing potential candidates from labor pools competitors have overlooked or ignored. Indeed, employees with disabilities compose “one of the largest underutilized labor pools” (Schur et al., 2014, p. 594; see also Kruse, Schur, & Ali, 2010; Kulkarni & Kote, 2014; Lengnick-Hall et al., 2008). However, in spite of this potential, managers consistently report that they find it difficult to attract qualified applicants with disabilities (Domzal et al., 2008).

To ensure that qualified individuals with disabilities are in the applicant pool, the recruitment process itself should not create barriers (Stone & Williams, 1997). In reality, the application process itself often inadvertently discourages participation. Bruyère et al. (2005), for instance, found that many electronic job boards and company websites have poor accessibility features and are not perceived as particularly welcoming. We know from signaling theory (Connelly, Certo, Ireland, & Reutzel, 2011) that the chances of successful recruitment will be increased if employers advertise their position broadly and in a way that signals that the employer is diversity-friendly. For example, employers can specifically list disabilities along with other forms of diversity in their formal diversity policy statements and in recruitment materials. Interestingly, an analysis of the diversity statements at Fortune 500 companies revealed that fewer than half included people with disabilities in their description of diversity (Ball, Monaco, Schmeling, Schartz, & Blanck, 2005).

As noted by Kulkarni and Kote (2014), employers “adopt ‘inclusion’ as a guiding value, but they simultaneously need to signal what they mean by this inclusion” (p. 189). Indeed, managers’ hiring of people with disabilities is not predicted by managers’ own positive intentions and attitudes toward people with disabilities, but by the presence of formal disability hiring policies and training specifically focused on hiring and retaining workers with disabilities (Araten-Bergman, 2016). Moreover, signs of commitment to the employment of people with disabilities start with top management establishing policies and ensuring that they are adhered to (Schur, Kruse, & Blanck, 2005). Thus, organization-wide and disability-specific diversity policies send the right signals to applicants and to hiring managers. In turn, these signals help employers increase the probability that their applicant pools contain qualified applicants *and* that those applicants are actually selected.

#### Practical Implications and Research Directions

In summary, adopting an inclusive approach begins before the hiring stage. Employers may wish to review their recruitment practices to ensure that they are not inadvertently dissuading applicants with disabilities from pursuing job openings. Managers should ensure that recruitment processes (e.g., online application portals) and messages do not act as barriers to possible applicants with disabilities. In this respect, employers can think about the implicit and explicit messages they send to potential applicants and whether those messages signal an inclusive climate (Connelly et al., 2011). Inclusive hiring practices also have positive implications for corporate reputation. Indeed, consumers evaluate organizations that hire people with disabilities more favorably than those that do not, and they prefer patronizing those organizations (Siperstein, Romano, Mohler, & Parker, 2006).

Recruitment efforts can be expanded if organizations proactively partner with vocational agencies and community-based organizations that specialize in supporting the employment needs of people with disabilities. These agencies play a key role in facilitating a successful employment relationship, by introducing hiring managers at the organization to the job applicant, assisting with the accommodation process, if needed, and troubleshooting post-hire challenges, if any (Hernandez et al., 2008). Although the assistance provided by these partners is often free, many employers are either unaware or do not make use of these and similar resources (Domzal et al., 2008).

In the US, services that address recruitment concerns include the aforementioned JAN and EARN. Of note, EARN provides employers with guidelines for building inclusive workplaces through their “Inclusion@Work” modules, which include advice on where and how to best recruit workers with disabilities, as well as free webinars on recruitment strategies. Advice on recruitment best practices is also typical assistance provided by nonprofit organizations, such as the National Organization on Disability. Importantly, resources for small businesses are also available from EARN. Finally, lists of recruitment support services are available to employers interested in broadening their searches to include people with disabilities; many of these services include the option of free job postings. Examples of these lists are available from EARN and in a Government of Canada publication about recruiting people with disabilities. These lists of recruitment support services may be particularly appealing to managers in smaller organizations or to those in organizations with smaller HR departments. We remind readers that the resources mentioned above are listed in Table 1.

More research is needed to understand the managerial and organizational barriers to effective recruitment of people with disabilities. For example, applying the theory of planned behavior (Ajzen, 1991) to study managers’ hiring intentions and decisions could help elucidate where some of these barriers reside. As Domzal et al. (2008) indicate, some managers report that recruiting applicants is difficult. This may be particularly true of small business owners, who have fewer organizational resources to recruit employees with disabilities (e.g., they may not have access to an HR department to support their recruitment efforts). These fewer resources would likely translate into lower perceived behavioral control, which would lead to lowered intent to proactively recruit applicants with disabilities, and a lowered probability of acting on these intentions, if they exist. Similarly, managers’ subjective norms can be influenced by competitors’ practices and industry norms. It would be useful to empirically assess how success stories of businesses that have recruited and hired inclusively affect other managers’ decisions to engage in similar practices. We return to the importance of success stories in concern 9.

### Concern 3: the Attractiveness of Job Openings to People with Disabilities

It has been suggested that even if people with disabilities eventually make it into applicant pools, hiring managers might incorrectly assume that these applicants do not want challenging careers or assignments (Perry, Hendricks, & Broadbent, 2000; Wilson-Kovacs et al., 2008). Worse, one prejudice that continues to affect people with disabilities is that they are perceived to not want to work at all (Hemphill & Kulik, 2016). These biases permeate decisions in all phases of the employment cycle.

However, the belief that people with disabilities do not want to work is demonstrably false. As reported by the National Organization of Disability (2004), over 60% of working-age people with disabilities in the US not currently employed would prefer to be employed. More recently, based on a nationally representative survey, Ali, Schur, and Blanck (2011) reported that the proportion of unemployed Americans with disabilities who would like to work is actually closer to 80%. This figure is no different for unemployed individuals who do not have a disability. Furthermore, people with and without disabilities attach the same significance to work-related outcomes such as job security, income, promotion opportunities, having an interesting job, and having a job that contributes to society (Ali et al., 2011).

Overall, there are more similarities than differences in terms of the types of positions to which workers with and without disabilities are attracted. One difference, however, is that people with disabilities may evaluate the attributes of the job (such as hours promised, benefits provided) vis-à-vis regulations surrounding their government-provided disability benefits (Fabian, 2013). A further difference is that people with disabilities may prefer government positions to private organizations, possibly because such jobs are perceived to provide better health benefits, more accommodations, and lower likelihood of discriminatory employment practices (Ali et al., 2011; Jans et al., 2012). In the US, Section 503 of the Rehabilitation Act (1973, as amended, 29 U.S.C. § 793) might also encourage employees with disabilities to look for positions with federal contractors or subcontractors.

Moreover, workers with disabilities often benefit from flexible work arrangements, especially if they face transportation barriers to get to work (Schur, 2003; Schmidt & Smith, 2007). Telework has been shown to be a facilitator of employment, a finding not limited to workers with mobility impairments (see Lidal, Huynh, & Biering-Sørensen, 2007 for a review; Jetha, Bowring, Furrie, Smith, & Breslin, 2018). Furthermore, workers with disabilities might be more likely to take part-time or contingent jobs: in Australia, there has been an upward trend in part-time employment for people with disabilities (Australian Bureau of Statistics, 2016), and in EU countries, people with disabilities are more likely than those without to be employed part-time (26 vs. 18%) except in Austria, where the rate is the same at 23% (Eurostat, 2017). While these figures show differences in employment type *held*, Ali et al. (2011) found that flextime is not a differentiating factor in the type of job *sought* in comparing people with and without disabilities in the US. Similarly, in Canada, the majority of people with disabilities seeking employment are able to work full-time (Till, Leonard, Yeung, & Nicholls, 2015). It is likely that many people with disabilities who are working part-time would have preferred full-time employment if it were available to them, which is also the case for workers without disabilities (Thorsteinson, 2003). Modified or flexible hours, are, however, one of the most common accommodations needed by both job seekers and employed workers (see, e.g., Till et al., 2015 for Canada, and Statistics Sweden, 2017 for Sweden; Jetha, Bowring, Furrie, et al., 2018).

#### Practical Implications and Research Directions

What the above results suggest is that, just as for people without disabilities, what attracts an employee to an organization is a matter of personal preferences and perceived fit (Chapman, Uggerslev, Carroll, Piasentin, & Jones, 2005). Thus, workplaces offering supportive employment practices for *all* employees will be able to facilitate employment for people with disabilities (Kaletta, Binks, & Robinson, 2012; Schur, Kruse, Blasi, & Blanck, 2009), thereby attracting and retaining talented workers who might otherwise exit the workforce. For example, the EARN Inclusion@Work modules and webinars (see Table 1) offer advice on building inclusive work cultures, which can be departure points for discussion during organizational strategy meetings. In this vein, organizations could explicitly include disability in their diversity and inclusion statements. As noted above, disability is often left out of these statements (Ball et al., 2005; Colella & Bruyère, 2011). Explicitly including disability in formal diversity statements and policies, and day-to-day practices that implement them, can help organizations move beyond adopting a compliance-based perspective that simply seeks to meet legislative requirements (Lengnick-Hall et al., 2008). Communicating information about these practices to internal and external stakeholders is critical because they convey core values upon which the organization’s culture is built. This communication reinforces perceptions of positive organizational climates within organizations and promotes a positive organizational image to external observers, such as customers, investors, and future employees. Supporting these initiatives, EARN provides advice on how to express and communicate a commitment to the inclusion of people with disabilities across the organization, something that can be implemented regardless of organization size.

While these resources exist to help organizations become more attractive to employees, more research would be beneficial. For instance, does the finding that organizations are more attractive to minority applicants when they share the demographic characteristics (e.g., gender) of recruiters and interviewers (see Avery, McKay, & Volpone, 2012, for a review) extend to applicants with disabilities? This is an important question given the invisible nature of many disabilities, as discussed in concern 1. Furthermore, it is important to determine when these initiatives lead to long-term employment. This research is necessary given that recruitment messages may not translate into long-term positive outcomes for applicants if the organizational practices are not supportive of workers with disabilities, a point argued by McKay and Avery (2005) in the context of recruiting members of racial minority groups.

## Employee Selection

### Concern 4: the Qualifications of Applicants with Disabilities

Once applicants are recruited, the next phase of the employment cycle is to process the applicant pool and make hiring decisions. At this stage, managers must assess the extent to which applicants’ personal characteristics (e.g., job-relevant knowledge, abilities, and skills) fit the qualities demanded by the job, and then use this information to make choices between applicants. A concern here is that managers sometimes believe that “people with disabilities can’t do the basic functions of the jobs they apply for” (Kaye et al., 2011, p. 529; see also Kulkarni & Kote, 2014). The very nature of how workers with disabilities are labeled emphasizes a *lack* of ability, which is in contrast to the nature of the role that all workers are expected to fulfill in organizations (Jammaers, Zanoni, & Hardonk, 2016; Baldridge, Beatty, Böhm, Kulkarni, & Moore, 2018). We address two forms of this concern below.

In some cases, the concern is specific, such as a fear that disabilities would prevent applicants from performing physically demanding tasks if they were hired (Gröschl, 2013; Lengnick-Hall et al., 2008). This concern may reveal managers’ underappreciation of applicants’ abilities. For example, in a study on employees with spinal cord injuries, Sinden and Martin Ginis (2012) found that many employees were performing jobs that exceeded what would have been “typically” expected of a person with this injury. The concern surrounding whether applicants with disabilities can perform physically demanding tasks may also reveal a lack of appreciation of the diverse nature of disabilities (Baldridge et al., 2018). In any event, physical abilities should only be used to predict future job performance when a job analysis determines that these human abilities are, in fact, necessary to perform critical job tasks. Even in industries like hospitality and tourism, in which some positions require mobility (e.g., housekeeping), Gröschl (2013) finds that many types of disability have no impact on employees’ ability to complete long shifts. Indeed, he argues that “by using selection methods that provide strong predictive validity of future job performance and matching [an employee’s] competencies with the job requirements, managers can ensure that an employee’s disability has no effect on his or her performance” (p. 121). Accommodations can be important here, and we discuss this topic in concern 6.

In other cases, the concern is broader, such that managers stereotype applicants with disabilities as lacking skills in general (Dovidio, Pagotto, & Hebl, 2011; Gröschl, 2013; Kaye et al., 2011; Lengnick-Hall et al., 2008). This concern is in line with the stereotype content model (Cuddy et al., 2009; Fiske, Cuddy, Glick, & Xu, 2002), which shows that people with disabilities are viewed as having high warmth (e.g., friendly, good-natured) but low competence (e.g., incapable, unskilled). In other words, managers may like these individuals but would not necessarily see them as hirable. A similar concern regarding negatively biased performance expectations for people with disabilities has also been discussed in the literature (Colella, DeNisi, & Varma, 1998).

Evidence from large-scale government surveys about the labor market characteristics of people with disabilities addresses this concern. For example, Ali et al. (2011) used the US 2006 General Social Survey to compare unemployed adults with and without disabilities. Ali et al. (2011) found no difference across groups on important markers of employability, such as the likelihood of ever having had a job that lasted for more than 1 year, being currently unemployed because of dismissal, or reporting their last job as being at the managerial level. When average differences between people with and without disabilities emerged, they were related to levels of formal education. For instance, unemployed individuals without disabilities had, on average, less than one additional year of education than those with disabilities.

The 2015 American Community Survey (US Census Bureau, 2015) includes additional information on the levels of education of people with and without disabilities. While the proportion of individuals with some college or an associate’s degree is virtually identical across groups (27.3 vs. 29.4% for individuals with and without disabilities, respectively), the groups differ on earned Bachelor’s degrees or higher (16.2 vs. 33.9%). The 2012 Canadian Survey on Disability (Arim, 2015) reports similar figures. Educational attainment is similar when considering high school degrees (80 vs. 89% for individuals with and without disabilities, respectively), and a difference emerges when considering university certificates or Bachelor’s degrees (14 vs. 27%). Australian figures (Australian Bureau of Statistics, 2016) report a gap between groups when considering Year 12 diplomas (41.0 vs. 62.8% for individuals with and without disabilities, respectively) and Bachelor’s degrees (17.0 vs. 30.1%). In general, the gap in education appears to grow across education levels. However, although many jobs require a college education, many do not. Thus, the differences in educational attainment do not fully explain the differences in employment rate (and employability) in census data (see, e.g., Lauer & Houtenville, 2017).

#### Practical Implications and Research Directions

Taken as a whole, these findings suggest that for many jobs, individuals with and without disabilities would likely present similar applicant profiles in terms of education; yet, these applicant groups fare differently. Employers concerned about qualifications may wish to proactively partner with local community colleges, vocational institutes, or universities, many of which offer partnership opportunities with, and assistance for, employers (see, e.g., the University of Guelph and the University of Washington, listed in Table 1). The advantage with such partnerships is that employers can first determine the educational program (e.g., degree type) that would supply the candidates with the right knowledge and skills, and, second, proactively recruit candidates with disabilities. Starting with internships, known as Co-Op placements in Canadian colleges and universities, might be appealing to employers who have no previous experience with employing graduates with disabilities, given that these programs have a built-in support system provided by the educational institution for all students. Heidkamp and Hilliard (n.d.) provide a comprehensive review of employer–educational institution partnership types and characteristics as well as a lengthy list of such partnerships in the US. An additional US-based service that has a mission to facilitate the employment of college graduates with disabilities and which provides support to both recent graduates and employers is the Workforce Recruitment Program.

Longitudinal research is required to track the experiences of students with disabilities as they enter the workforce. In particular, it would be useful to examine how being hired through a program specifically aimed at youth with disabilities affects new career entrants’ likelihood of requesting accommodations, and their eventual career trajectory within the organization (e.g., promotion opportunities). It has been established that workers at the beginning of their careers are less likely to request accommodations and face barriers related to the perceived cost of accommodations (Jetha et al., 2016; Jetha, Bowring, Furrie, et al., 2018). It would be important to establish if disclosure is facilitated if employers hire through the recruitment channels mentioned above, and, more importantly, if these new employees are protected from negative stereotypes (e.g., an accommodation for an invisible disability being perceived as entitlement) they may face.

### Concern 5: the Selection Process for Applicants with Disabilities

Another concern that has been noted is that some managers privately feel that applicants with disabilities complicate the selection process, inasmuch as “they [managers] can’t ask about a job applicant’s disability, making it hard to assess whether the person can do the job” (Kaye et al., 2011, p. 529), or managers may be worried about saying the wrong thing and being sued (Hernandez et al., 2008). Indeed, many employers acknowledge that they lack the necessary training at all stages of the employment relationship (Kaye et al., 2011; Wilson-Kovacs et al., 2008). More generally, managers may be unaware of selection best practices, even without considering disabilities (Rynes, Brown, & Colbert, 2002).

Interviews are the most common type of selection instrument (Poulakos, 2005), and employers might be anxious about ensuring that job candidates with disabilities have a positive interview experience. This intention is important, especially considering that only slightly over half of organizations taking part in a large Society for Human Resource Management (SHRM) survey report providing training to HR staff and supervisors on effective interviewing approaches for candidates with disabilities (Erickson, von Schrader, Bruyère, & VanLooy, 2014). It is therefore not surprising to find that interviewers negatively react to job candidates’ disabilities in an interview context (Hebl & Skorinko, 2005). There is also evidence that interviewers recall less information about interviewees who have a facial stigma (a scar or birthmark) and spend more time looking at the affected body part (Madera & Hebel, 2012). This effect is likely to be replicated for physical, cognitive, or sensory disability features, especially when those features are highly visible in an interview, such as a job candidate’s face, arms, and hands, and the use of a wheelchair, guide dog, or a white cane.

#### Practical Implications and Research Directions

In general, unless it is done in the context of customized employment,^3^ the selection process will not necessarily or automatically be different if someone with a disability applies for a job. In North American jurisdictions, employers are not entitled to ask applicants to list disabilities or health conditions, just as they are not allowed to ask about other protected information such as religion, national origin, or age (Canadian Human Rights Commission, 2007; U. S. Equal Employment Opportunity Commission, n.d.-b). Furthermore, employers should be clear about any necessary abilities required to perform the job at the beginning of the application process, and the need for these abilities should be demonstrated by a job analysis. If specific abilities are required to perform the essential duties of the job (that is, they are considered Bona Fide Occupational Requirements or Qualifications), employers may ask *all* applicants about their ability to carry out these essential duties. The question should be phrased as “How would you perform this required task?” Of note, employers should not ask this question only of applicants they suspect of having a disability. Using a consistent approach to selection, such as structured interviews, is important. Indeed, structured interviews can help prevent biased decisions against candidates with a history of disabilities (Reilly, Bocketti, Maser, & Wennet, 2006). EARN provides advice on effective interviewing, the main theme being to focus on abilities rather than *dis*abilities. An interview toolkit developed by Hire for Talent focuses on ensuring that interviewers ask *legal* questions, language and communication style during interviews, and other relevant communicative information such as how to greet candidates with different types of disabilities (e.g., a mobility impairment or a visual impairment). Similar interview advice is available from AND in Australia (see Table 1).

Overall, the selection process itself should not be a barrier to employment, so accommodations should be made available when necessary. In this case, the selection process will be different for candidates with disabilities. For instance, if the selection process called for a passing score on a “paper and pencil” test of safety rules and materials handling, then an easy way to accommodate candidates who have difficulty using a handwriting tool would be to ask the questions in a different testing format. The employer and the candidate might work together to determine whether using a keyboard, scribe, or dictation software would be most appropriate for this test. Employers are rightly concerned with the fairness, accuracy, and costs associated with any selection process, so they should be transparent with all applicants regarding the process and format of any employment tests required, choose tests with predictive validity for the target job, and minimize costs where possible. Medical assessments related to essential job duties should only be conducted after a conditional offer of employment has been made (U.S. Equal Employment Opportunity Commission, n.d.-a).

While managers may be concerned about the inconvenience of having to accommodate applicants during the selection process, they may also wish to think about applicants’ reactions to the selection process. Indeed, applicants’ reactions to selection methods and processes influence organizationally relevant outcomes, such as their perceptions of (un)fairness of treatment, process, or decision; their intent to accept an eventual job offer; their perceptions of organizational reputation; and willingness to recommend the employer to others (see Hausknecht, Day, & Thomas, 2004; Truxillo & Bauer, 2011). Future research could focus on how applicant reactions are shaped specifically for applicants with disabilities. For example, it is likely that the employer’s willingness to engage in accommodation discussions influences perceptions of justice, but how and why is each type of justice (procedural, distributive, and interactional) affected?

### Concern 6: the Cost of Accommodations^4^

In many jurisdictions around the world, laws (e.g., the ADA in the US) specify that it is illegal to not provide reasonable accommodations, in particular if applicants or employees disclose a disability by requesting accommodations. However, managers often have reservations concerning the perceived value of accommodating employees with disabilities (Gold et al., 2012; Hernandez et al., 2008; Kaye et al., 2011; Lengnick-Hall et al., 2008; see also Domzal et al., 2008). In essence, the issue (and source of discomfort) is that productivity benefits might not be enough to justify costs to the business (Hernandez, Keys, & Balcazar, 2000; Hernandez et al., 2008; see also Gaunt & Lengnick-Hall, 2014). This apprehension is aptly summarized by a respondent in a study on barriers to career advancement for people with disabilities who said: “Unlike other diversity families [….] disabled people come with a price tag – to remove doors to let in a wheelchair costs money” (Wilson-Kovacs et al., 2008, p. 711).

Managers’ beliefs and apprehensions around accommodation costs are frequently overstated. For example, JAN (2018) has tracked accommodation costs incurred by their clients since 2004. Accommodation costs of $0 (e.g., giving an employee access to park in more than one lot; Wilson-Kovacs et al., 2008) were reported by 59% of survey respondents. The majority of the other respondents reported a one-time cost less than $500. This figure is consistent with other reports on accommodation costs (e.g., Fredeen, Martin, Birch, & Wafer, 2013; Kaye, 2001; Lengnick-Hall, Gaunt, & Collison, 2003; Schur et al., 2014; Solovieva, Dowler, & Walls, 2011; Solovieva & Walls, 2013). Furthermore, the earlier the organization provides accommodations, the lower the costs; a lack of early attention to accommodation needs can lead to higher costs over time (Gardner & Johnson, 2004).

It is also worth noting that accommodations are frequently requested by workers *without* disabilities (Schur et al., 2014). This is important because the cost to accommodate employees with disabilities is no more than the cost to accommodate those without disabilities (Sabat et al., 2014; Schur et al., 2014). For example, from an organizational perspective, there would be no difference in cost in offering scheduling flexibility to an employee who travels via adapted transportation, to an employee with young children, or to an employee who is training for the Olympics, all of whom may require flexibility in the mornings or afternoons. Indeed, accommodations that would have been put in place for employees with disabilities (e.g., telework) benefit employees without disabilities as well; thus, not only are the accommodations less stigmatizing for one group of employees, but they may well help to foster a flexible and inclusive climate (cf. Connelly et al., 2011).

Importantly, accommodations are usually cost-effective. For example, Kaye (2001) estimates a $40 savings for every $1 invested in accommodation. Schur et al. (2014) found that the monetary benefits related to accommodation were “equal or exceed[ed] the costs in over two-thirds of cases, although it is difficult to quantify many of the benefits” (p. 614–615) especially in terms of positive spillover effects on coworkers’ and managers’ attitudes and overall organizational productivity. Similar benefits are reported by JAN (2018): 89% of the survey respondents indicated that the accommodations helped retain employees (see also Schmidt & Smith, 2007; Solovieva et al., 2011; Solovieva & Walls, 2013), 72% reported improved employee productivity, 56% noted increased employee attendance, and 38% reported observable saving in workers’ compensation and other insurance costs.

Often, the costs of accommodating are much lower than those incurred by *not* providing accommodations: the cost to hire a new employee (e.g., to replace an employee with a disability) typically exceeds $500 (O’Connell & Kung, 2007). The tax credits and financial incentives available in some jurisdictions (see Table 1 for some examples) can also be used to offset costs associated with accommodating and retaining workers with disabilities (Domzal et al., 2008; Mik-Meyer, 2016). Finally, and perhaps more importantly, providing accommodations to all employees regardless of disability status can have clear benefits in terms of improved perceived organizational support, commitment, job satisfaction, employee morale, and decreased turnover; of course, the benefits are greatest when coworkers are supportive of accommodations (Schur et al., 2014; Solovieva et al., 2011; see also Fredeen et al., 2013; JAN, 2018).

#### Practical Implications and Research Directions

Managers might find the accommodation process both uncomfortable and intimidating, especially if they are inexperienced. Complicating matters further is the fact that the accommodation process can require multiple attempts before the right accommodation is found. In an analysis of Canadian arbitration cases, Williams-Whitt and Taras (2010) found that almost half of workers with disabilities required more than four attempts to accommodate their disabilities, because the new tasks assigned were initially too difficult, there were unanticipated challenges to the workflow (i.e., impact on other employees), or the employee was re-injured. As noted earlier, accommodations are typically not expensive; however, they must be implemented appropriately and be tailored to the person.

Because of the central nature of accommodations to the successful hiring and employment of people with disabilities, interested employers may turn to JAN (see Table 1). One key resource offered by JAN is a searchable database of accommodation ideas, searchable by disability, limitation, occupation, and other features. JAN also offers free one-on-one consulting services for accommodation support for employers in the US. The no-cost and comprehensiveness of JAN’s resources can be particularly appealing to smaller businesses that do not have HR departments. The Equal Employment Opportunity Commission also has information on reasonable accommodation for small employers. Additional encouragement comes from reading success stories from businesses of all sizes provided by EARN and the US Chamber of Commerce. Often, industry-specific evidence can be more convincing to employers than research evidence.

As discussed earlier, both employers and employees with disabilities report a need for better training for managers and HR specialists to increase their knowledge of best practices relating to employees with disabilities. This is particularly important for accommodations, given that they are often the crux of successful long-term employment. This training would permit the focus to shift from legal compliance, to a focus on helping everyone learn to think more creatively and constructively about accommodations, and to see the many benefits of accommodations and inclusive workplace practices (Kaye et al., 2011; Schur et al., 2014; see Fredeen et al., 2013, and Kaletta et al., 2012, for useful suggestions). For example, the Canadian Human Rights Commission offers a series of five webinars on the accommodation process and a set of case studies to help employers think through complex accommodation cases.

More research is necessary on how to make the accommodation process more effective. For example, it is possible that an iterative, or “early and often” accommodation process, in which initial changes are made and then revisited regularly is actually more cost-effective in the long run, because employers would be able to meet employees’ immediate needs quickly and then employees could suggest modifications on an as-needed basis as duties change and new technologies emerge. In contrast, a centralized or more rigid process in which employees are expected to request all possible accommodations at the beginning of their employment (i.e., “one and done”) and provide extensive documentation may in fact be more unwieldy and expensive. A utility analysis would provide evidence of the best approach.

## Social Integration

### Concern 7: the Impact of Workers with Disabilities on Coworkers

Once in the organization, the next overarching phase of the employment cycle concerns initial and ongoing adjustment to task and social realities. Thus far, our emphasis has been on the former. However, when it comes to workers with disabilities, some managers have expressed concerns about their ability to fit in socially and concerns that these individuals might adversely impact other coworkers (who presumably do not have a disability). Kaye et al. (2011) found that managers were “concerned about attitudes of co-workers toward the person with a disability” (p. 529; see also Domzal et al., 2008), while Lengnick-Hall et al. (2008) found that some managers were concerned about the negative impact on morale (see also Gaunt & Lengnick-Hall, 2014). In particular, managers may be concerned that employees with disabilities will be disruptive to team functioning, or that coworkers without disabilities will perceive accommodations as unjust (e.g., fewer responsibilities for the same pay, access to better equipment; Colella, 2001; Colella, Paetzold, & Belliveau, 2004; Gold et al., 2012; Schur et al., 2005; Travis, 2008). Similarly, managers may fear that coworkers will resent having to work more to compensate for the anticipated low productivity of the person with disabilities or that they will perceive the work as being unfairly redistributed if jobs are changed following accommodations (Kosny et al., 2013).

Fundamental to these assumptions is the notion that a worker who has a disability will (a) be identifiable as such and (b) have noticeably lower performance or ability than employees without disabilities. As discussed above, the nature of many disabilities is such that coworkers will be unaware of someone’s disability. Also, in countries that have laws regarding confidentiality, managers cannot disclose accommodations made to other employees or discuss an employee’s disability (Santuzzi et al., 2014), though some accommodations (like schedule flexibility) may be apparent to others.

Aside from these assumptions, what remains are the perceptions among some managers that employees without disabilities will resent accommodations that are provided to those who need them. The evidence suggests otherwise. Indeed, Solovieva et al. (2011) found that a benefit of making accommodations was improved interactions between employees with disabilities and their coworkers and increased overall company morale. Furthermore, because both employees with and without disabilities may require accommodation (Schur et al., 2014), accommodations will help productivity, increase commitment, decrease turnover, and can have positive effects on all coworkers’ attitudes. Indeed, accommodations send important and positive signals to employees by showing that the organization values the contributions of its employees and cares about their well-being. Signaling organizational support is not trivial, inasmuch as these perceptions lead to positive work experiences, such as affective commitment (e.g., Kurtessis et al., 2017). In turn, affectively committed employees are more likely to remain with their organization and more likely to exhibit a wide range of positive work outcomes, such as job performance and organizational citizenship behavior (Allen, 2016).

Providing reasonable accommodations is required by law in many countries. However, to the extent that the manager or organization is perceived to be proactive or providing assistance above and beyond what is legally required, employees are likely to respond positively. A parallel is found in the broader HR literature; employee attributions about the reasons for certain HR practices, such as the perceived authenticity of diversity initiatives (Smith, Morgan, King, Hebl, & Peddie, 2012), influence employee attitudes and behaviors (Nishii, Lepak, & Schneider, 2008). Thus, after observing how an accommodation greatly benefited a team member with a disability, others might come to interpret these practices as stemming from genuine managerial support for employees rather than mere legal compliance.

Rather than negatively affecting workplace morale (see Solovieva et al., 2011), there is evidence that employees with disabilities will have a positive effect on the organizational attitudes of their coworkers. This influence goes beyond any superficial stereotypes of individuals with disabilities being “inspirational” or interpersonally warm (Stone & Colella, 1996). Employees with disabilities exhibit stronger feelings of affective commitment to their organization relative to their counterparts without disabilities (Hernandez et al., 2008; Kulkarni & Gopakumar, 2014). This can further benefit organizations if emotions are transferred to others (Barsade, 2002). Finally, Nittrouer, Trump, O’Brien, and Hebl (2014) have argued that the act of voluntarily disclosing an invisible disability can have a beneficial effect on relationships with coworkers. Because disclosure increases personal risk and makes one more vulnerable, the message conveyed to coworkers is one of trust—effectively acting “as a catalyst to kick start social change” and evoking protective motives within the group (p. 237). To the extent that employees with disabilities are known in the workplace, their attitudes and day-to-day behaviors should provide a source of informational and social cues to other members, which should, in turn, influence the attitudes and behaviors of their coworkers.

#### Practical Implications and Research Directions

In summary, while managers may believe that workers with disabilities will have a negative impact on their coworkers, the empirical evidence suggests otherwise. As discussed earlier, the concern may stem from a lack of knowledge. This unawareness can be remedied by training and development for managers and employees about working with people with disabilities, a service offered by many local organizations that support the employment of people with disabilities. For example, employers in Canada interested in finding local service providers can consult Hire for Talent, which lists providers by province. In the US, employers can consult EARN, which also lists service providers by state (see Table 1).

Managers’ concerns that coworkers who do not require an accommodation might resent those who do might be assuaged by efforts to change the discourse around accommodations. Indeed, equating accommodations solely with disabilities in an organization may contribute to the view of workers with disabilities as “different” or “difficult” (Kaye et al., 2011). Instead, organizations that support the needs of all employees, regardless of disability status, may fare better (Goetzel et al., 2016; Travis, 2008; see also Schur et al., 2014). By adopting a broader perspective on accommodation, more as a core organizational value, adjustments will, over time, be viewed as instrumental in achieving person–job/person–organization fit. Moreover, accommodating the diverse needs of all employees (due to disability or not) should help to change a negative organizational discourse on disabilities to one that recognizes that everyone benefits from inclusive workplace practices. A positive approach to this discussion is exemplified by a leading law firm in a major Canadian city, in which hiring managers ask of all candidates “What do you need to make yourself more successful in our firm?” (Fredeen et al., 2013, p. 13). This approach sets the stage for an employment relationship focused on respect, open communication, and success predicated on abilities (rather than *dis*abilities). Reflections on inclusive workplace policies focused on meeting the needs of all employees, including an example of a policy statement on accommodations for all employees, are available from Hire for Talent. Additional ideas on creating an inclusive workplace are offered by the aforementioned Canadian Human Rights Commission webinars.

Research specifically on the effects of including workers with disabilities in a team or work unit is necessary. For example, a recent meta-analysis on the impact of diversity on team performance (Bell, Villado, Lukasik, Belau, & Briggs, 2011) focused on age, sex, and race as demographic variables, but disability status was not considered. A relevant distinction made in this meta-analysis was that of teams focused in intellectual tasks (e.g., negotiation, design) versus those focused on physical tasks (e.g., production). It may be that the type of disability (visible, invisible, physical, intellectual) interacts with the type of team in question.

### Concern 8: the Organizational Integration of Workers with Disabilities

Related to the previous concern, some managers report that they are uncertain how to approach social integration of employees with disabilities within the work unit and broader organization (Kaye et al., 2011). This is an important consideration. The successful adjustment of employees with disabilities is determined, in part, by organizational culture and the extent to which diversity and inclusive work practices are valued and enacted by both leaders and coworkers (Schur et al., 2009; Schur et al., 2005; Vornholt, Uitdewilligen, & Nijhuis, 2013).

However, employees with disabilities report greater experiences of subtle discrimination, such as being excluded from informal gatherings, or being ignored in meetings as compared to employees without disabilities (Naraine & Lindsay, 2011; Snyder, Carmichael, Blackwell, Cleveland, & Thornton, 2010). Often, fostering a climate of inclusion requires coworkers to be considerate (e.g., introducing oneself to a blind or low-vision coworker, confirming that events held at offsite locations are accessible). Ensuring that employees with disabilities do not experience subtle discrimination is critical given that subtle discrimination is as damaging to those who experience it as are more overt forms (Jones, Peddie, Gilrane, King, & Gray, 2016). Furthermore, having experienced discrimination in the past leads to anticipated future discrimination, which in turn leads to workers being more likely to engage in concealing (e.g., hiding symptoms) and compensatory behaviors (McGonagle & Hamblin, 2014).

Of course, this discussion assumes that the employee with a disability has been with the organization for some time. Indeed, it is not unusual for employees without disabilities to develop or acquire a disability later in their careers (Baldridge & Kulkarni, 2017). However, for new employees, ensuring that the socialization process provides the right opportunities for integration is important. Organizational leaders’ behavior vis-à-vis employees with disabilities will set the tone for coworkers’ own behaviors; if supervisors do not behave in ways that demonstrate acceptance of the newcomer, it is unlikely that colleagues will (Kulkarni & Lengnick-Hall, 2011; Schur et al., 2005). Coworkers also help in the socialization of employees with disabilities, by engaging in cooperative behaviors (e.g., introducing new employees to colleagues), helping them with task-related functions, and acting as mentors. The visible presence of other coworkers with disabilities also helps with socialization (Kulkarni & Lengnick-Hall, 2011). Further, Naraine and Lindsay (2011) suggest that socialization could also be devoted to meeting the individual needs of employees, such as allowing extra time for newcomers who are blind or have low vision to meet with sighted colleagues to get to know the colleague with a disability “as individuals” (p. 401). Thus, the spirit behind any activity should be to foster and cultivate social inclusion for all employees.

#### Practical Implications and Research Directions

In summary, the organizational integration of workers with disabilities is an important part of developing an inclusive organizational culture. Organizations of all sizes that are interested in benchmarking their current practices can use the free and confidential “Disability Employment Tracker” offered by the National Organization on Disability (NOD; see Table 1). The survey and its associated resources help organizations reflect and develop action plans centered on several key business processes, including climate and culture. One way that inclusive organizations support workers with disabilities is through the creation of employee resource groups, a practice recommended by NOD and EARN. Employee resource groups are encouraged by EARN because they bring important business outcomes such as increased retention, performance, and commitment of workers, as well as help train those who do not take part in the group on disability-related issues, among other benefits. Of note, Von Schrader et al. (2014) found that the presence of employee resource groups was a facilitator of disclosure, especially among employees with less apparent disabilities. Because of the benefits associated with employee resources groups, EARN has developed practical guidelines to support organizations in establishing these groups. This source includes advice on all aspects of an employee resource group lifespan, from creating it to measuring its success. The important role of employee resource groups is also highlighted by EARN in its Inclusion@Work initiative, and specifically in the module on inclusive business cultures. To be sure, employee resource groups can be more easily established in large organizations that employ many workers with disabilities. However, some of their benefits can be reaped in smaller organizations as well, if employees are encouraged to participate in industry-specific groups that encompass several employers. Local Chambers of Commerce may be useful starting points to connect with other smaller organizations.

Despite the promise afforded by the creation of resource or affinity groups, research is needed to determine how they may be organized to provide the most benefit to employees. It is not yet clear if expanding the membership to include “allies” is useful because it enables employees to participate without disclosing their own status, or if it is counterproductive because it undermines the focus on providing a forum for the voices of people with disabilities. Furthermore, even workers whose disabilities are apparent may avoid situations in which they may be identified primarily as someone with a disability rather than as an industry professional or expert in their field. Indeed, not all workers wish to espouse a disability identity instead of, or in addition to, other relevant identities in the workplace (e.g., gender, race, occupational identity; see Santuzzi and Waltz (2016), for an excellent discussion of the topic of disability identity). The fear of being stigmatized may weigh heavily in reflections surrounding which identity(ies) to espouse in a work context. The issue of stigmatization has been studied comprehensively in the literature on affirmative action programs in employment contexts, in the US and in other countries that have similar policies (see Harrison, Kravitz, Mayer, Leslie, and Lev-Arey (2006), for a thorough review).

Research is also needed on how internal messaging, often from the HR department, affects how workers with disabilities are perceived and treated by their colleagues. Many companies publicize the hiring of workers with disabilities as part of corporate social responsibility programs. Although it is intended to be positive, this emphasis may have the unintended effect of suggesting that these employees were hired because of their disability, not because of their expertise or productivity. Similarly, it is not unusual for companies to emphasize in their materials that people with disabilities are “no different” from every other employee. Again, while well-intentioned, this emphasis may imply that (a) difference is problematic and (b) workers with disabilities are not different enough to actually require accommodations. Experimental studies that compare the effects of these types of internal messaging campaigns to those that provide specific information on employee rights and how to access accommodations would provide useful guidance to organizations that seek to create a more inclusive work environment. The work on multiculturalism versus color-blind approaches to diversity in the context of research on ethnic diversity would be particularly informative for this line of inquiry. Indeed, color-blind approaches, which downplay group differences, have been shown to be less effective than multicultural approaches, which suggest that we should consider membership to different groups as being important, and that differences should be celebrated rather than ignored (Richeson & Nussbaum, 2004). Similar dynamics may be at play in the context of disability-related diversity.

## Performance Management

### Concern 9: the Job Performance of Workers with Disabilities

An often noted concern of employers surrounds the job performance of workers with disabilities; employees with disabilities are presumed to be less productive than employees without disabilities (Lengnick-Hall et al., 2008; Stone & Colella, 1996; see also Domzal et al., 2008; Fredeen et al., 2013). Relatedly, employees with disabilities are perceived as having other performance issues, such as slowing down work (Hernandez et al., 2008), higher absenteeism and lateness (Gröschl, 2013; Hernandez et al., 2008; Kaye et al., 2011), or simply being less dedicated or dependable (Kaye et al., 2011) than employees without disabilities. In other words, employees with disabilities are sometimes perceived by managers as “problem employees” (Kaye et al., 2011, p. 529). These beliefs may be consistent with several negative stereotypes, such that workers with disabilities are perceived as weak, need assistance, need more supervision, or need too much training (Dovidio et al., 2011; Kaye et al., 2011). The persistence of these particular beliefs makes it difficult for managers who might otherwise encourage the proactive hiring of workers with disabilities; doing so is therefore presented as a charitable act that runs counter to organizational success or stakeholder value.

Dispelling the concern of low performance, Lee and Newman (1995) found that HR managers who had accommodated employees’ disabilities had rated the performance of 72% of these employees as average, above average, or excellent. More recently, Kaletta et al. (2012) analyzed productivity differences between employees with and without disabilities. They found that across 31 locations in three distribution centers, the difference in productivity for workers with and without disabilities was statistically insignificant in 18 locations. When there were productivity differences, employees *with* disabilities were more productive in 10 locations, while those without disabilities were more productive in three locations. Similarly, the industry report by Hernandez and McDonald (2007) found no differences in performance or need for supervision between employees with and without disabilities, the latter dispelling another concern expressed by managers (Kaye et al., 2011).

In instances in which workers with disclosed disabilities demonstrate lower performance than their counterparts without disabilities, it is important to ascertain the underlying reasons for this discrepancy. One possible reason for lower relative performance is that appropriate accommodation has not been provided or implemented (Gignac et al., 2015). For example, a data entry clerk with arthritis who does not have access to an ergonomic keyboard or mouse may require more time to complete tasks that involve typing; his or her performance would therefore be lower than it could be (and may be lower than that of employees without disabilities). As noted earlier, accommodations are typically not expensive; however, they must be implemented appropriately and be tailored to the person. Moreover, “as with any other employee, the failure of a person with a disability to meet certain performance standards can be caused by a wide range of factors that are not related to a person’s abilities, including motivation, unclear job requirements, and lack of organizational or managerial support” (Gröschl, 2013, p. 121).

Absenteeism and lateness have been highlighted as being particular concerns for managers (Gröschl, 2013; Hernandez et al., 2008; Kaye et al., 2011). Just as for performance concerns, there is evidence that workers with disabilities do not experience higher levels of lateness or absence in comparison to employees without disabilities (Kaletta et al., 2012; see also Fredeen et al., 2013). Indeed, Hernandez and McDonald (2007) found better or equal attendance records for workers with disabilities, except in organizations that also reported fewer accommodations. Finally, Kaletta et al. (2012) found that workers with disabilities had significantly lower turnover rates than their counterparts who did not have disabilities. Similar findings are summarized in a Canadian government report (Fredeen et al., 2013), revealing substantially lower turnover in a large hotel chain for employees with disabilities versus those without (6 vs. 52%).

#### Practical Implications and Research Directions

In summary, while managers may express concern that workers with disabilities would have lower job performance and greater incidence of lateness or absenteeism, the empirical evidence suggest otherwise. Not surprisingly, organizations that aim to increase the workforce participation of people with disabilities often rely on success stories when speaking with members of the business community. Interestingly, employers’ testimonials make it clear that their inclusive practices do not stem from charity but from business decisions (see Table 1). This perspective underscores that hiring and retaining workers with disabilities means that they, like all other employees, must be able to perform the job elements that a job analysis has demonstrated as essential. Thus, a person who uses a wheelchair would not be able to perform effectively as a lifeguard in a community center pool but could be hired and be successful in other roles in the center, depending on their training and interests, such as youth services coordinator or swim coach.

Business owners or managers who are new to employing workers with disabilities may find working with a specialized employment resource center to be helpful. Indeed, employment centers’ staff work with both the employer and the workers to ensure good performance and productivity and to find solutions if problems do arise. This strategy might be particularly appealing to owners of smaller businesses who have fewer resources (e.g., time) to devote to diagnosing the roots of performance difficulties (e.g., poor instructions, lack of proper resources, poor person–job fit). Often simple and inexpensive changes (e.g., written instructions) are required to remedy the performance difficulties, as discussed in the previous section on accommodations. Research is necessary to fully explore the role of these employment resource centers, and the instances in which they may be most useful to employers. It may be that organizations that proactively seek out the aforementioned support provided by these centers are more successful in avoiding the negative and ableist stereotype of lower productivity faced by employees with disabilities (Jammaers et al., 2016).

### Concern 10: the Occupational Health and Safety Behaviors of Workers with Disabilities

Safety is an important work outcome when it comes to organizational effectiveness. Thus, it is interesting that some managers fear that workers with disabilities introduce the potential for safety problems and higher accident rates (Lengnick-Hall et al., 2008; see also Domzal et al., 2008; Gaunt & Lengnick-Hall, 2014). The evidence suggests these concerns are likely to be unfounded. An industry report (Du Pont, 1990) suggests that workers with disabilities have equal—if not better—safety awareness and records than those without disabilities and that their safety awareness positively influences other organizational members. Another industry report found that while some employers reported more claims for employees with disabilities, the authors indicate that these results are limited because not all employers surveyed reported these figures (Hernandez & McDonald, 2007). The divergent findings may be explained by a recent study which suggested that workers with disabilities are more likely to report all injuries, no matter how minor (i.e., they follow reporting guidelines exactly); when considering only more serious injuries, their numbers are much lower (Kaletta et al., 2012). Furthermore, employees with disabilities may be more vulnerable to work injury but not because of their specific behavior; rather, they may be more vulnerable to an unsafe environment around them (Breslin, Lay, Jetha, & Smith, 2018).

Unfortunately, when workers with disabilities incur a workplace injury, they may have a lengthier return to work process (Smith et al., 2014), though this process depends on several factors such as the nature of the injury, its interaction with the existing disability, employee age, and the supportiveness of the organizational climate. Indeed, Kaletta et al. (2012) report *less* time away from work due to accidents and lower workers’ compensation costs for workers with disabilities. Finally, accommodations and proactive management of disabilities can help reduce workers’ compensation and insurance costs (Gardner & Johnson, 2004; Solovieva & Walls, 2013).

#### Practical Implications and Research Directions

In summary, employers may be concerned that workers with disabilities introduce safety hazards in the organization or are more prone to injury than workers without disabilities. The evidence reviewed above suggests these fears to be generally unfounded: workers with disabilities are not more likely to injure themselves or others than those without disabilities. One area in which safety considerations may come into play is emergency situations, and it is sound business practice to ensure that emergency preparedness considers all employees’ needs. This may require having an individualized emergency response plan for each worker with disabilities (e.g., determining how a worker with a visual impairment or one who uses a mobility device can safely evacuate a building in case of fire). Furthermore, individualized plans may rely, with the employee’s consent, on assistance from one or more coworkers. These plans should be updated when the worker’s job environment changes (e.g., new location, new coworkers) and revised regularly to ensure they are still appropriate. In some cases, an individualized emergency preparedness plan is a legal requirement, as it is in the Canadian province of Ontario (see Micheelsen & Williams, n.d. for information). Advice on inclusive emergency preparedness is also offered by the AND (see Table 1).

As noted above, there is evidence that workers with disabilities are more conscientious about reporting even minor safety violations in the workplace (Kaletta et al., 2012). This is useful behavior that safety-conscious organizations generally seek to encourage. Research is necessary to determine why these individuals are more assiduous: possible factors include greater job insecurity, a greater appreciation for the consequences of unsafe behaviors, or greater exposure to complicated rules and protocols (e.g., from treatment or rehabilitation processes). In-depth surveys of matched samples of workers with and without disabilities would be important for this research.

### Concern 11: Disciplinary Action and Termination of Workers with Disabilities

Finally, we note in our review that many managers report being uncertain of how to take disciplinary action or fire a worker with disabilities who does not meet performance expectations, and they may be worried about legal consequences for mishandling this process (Lengnick-Hall et al., 2008; Kaye et al., 2011; see also Gaunt & Lengnick-Hall, 2014). However, for all employees, regardless of disability status, organizations should be proactive in managing (and documenting, as necessary) performance issues, providing training or accommodations where relevant, and providing clear performance expectations. If performance difficulties do arise, regular and immediate feedback is important regardless of disability status. Gröschl (2013) shows the benefits of immediate feedback that made use of factual and objective examples when employees with disabilities exhibited low performance. Importantly, termination due to poor performance might be considered discriminatory if the proper training or accommodations have not been provided.

When legal action does occur in the context of the ADA, the decisions most often favor employers (Lee, 2001). Furthermore, employees with disabilities report that legal action occurs typically after other attempts at receiving reasonable accommodation have failed and that legal actions are a result of a lack of organizational-level knowledge on how to best support the careers of people with disabilities (Wilson-Kovacs et al., 2008). Legal action is a last resort that is perhaps affected by some managers’ adherence to false stereotypes that view people with disabilities as entitled or asking for special treatment (Kaye et al., 2011). Indeed, some have argued that the vagueness inherent in accommodation laws and requirements can contribute to this stereotype (Wilson-Kovacs et al., 2008). Furthermore, managers often report knowledge gaps on accommodation best practices and understanding of the disability experience (Kaye et al., 2011; Wilson-Kovacs et al., 2008). Therefore, it is important for managers and people with disabilities to work on accommodations as partners and allies. Accommodations are most effective when all parties work together as true partners, and when alternative strategies are pursued if initial attempts at accommodation are unsuccessful.

#### Practical Implications and Research Directions

Employers are often fearful of litigation, something that might be particularly concerning for small organizations, which do not benefit from the support provided by a legal department. Employers who are proactive in terms of understanding the legal context such as by visiting websites that provide information on employment laws in their jurisdiction and, more importantly, completing training courses either in person or online demonstrate goodwill and ensure that managers are aware of proper practices (see Table 1). Furthermore, seeking confidential assistance, such as the one provided by the ADA National Network, when in doubt or as soon as issues arise will ensure that any problems do not escalate. Small business owners might also benefit from joining business networks or societies dedicated to the inclusion of workers with disabilities. SenseAbility is one such organization in Canada, and Disabiliy:IN is one in the US. Businesses can also find other local champions of inclusive practices through local Chambers of Commerce. The ability to learn from peer organizations (e.g., similar sizes, industries), share success stories, and learn from one another’s failures may be particularly reassuring to small business owners.

Legal research on the factors that make workers with disabilities more (or less) likely to sue their employer would provide useful guidance in terms of how to prevent lawsuits. Similar research in the medical field has found that physicians who apologize to patients (or their families) for their medical errors actually reduce the likelihood of medical malpractice lawsuits, even though they are admitting liability (Ho & Liu, 2011). In the employment context, it would be useful to determine if any aspects of managers’ or coworkers’ behaviors (e.g., derogatory language, exclusion) are disproportionately associated with civil suits. Archival research that examines legal decisions would be especially useful.

## Conclusion

Workers with disabilities form one of the largest diversity groups in the workplace (Hyland & Rutigliano, 2013). Because of the high level of unemployment among people with disabilities, many have argued that they are insufficiently utilized as a labor pool and that employers will want to recruit from this pool to address the labor shortage caused by demographic shifts as the baby boomers retire and are replaced by fewer new entrants to the workforce (Lengnick-Hall et al., 2008; Kruse et al., 2010; Schur et al., 2014; see also Fredeen et al., 2013).

Yet, despite advances in diversity and inclusion practices in the workplace, the entry and progression of people with disabilities in the workforce remain problematic. Indeed, Lengnick-Hall et al. (2008) argue that “most employers hold stereotypical beliefs not supported by research evidence” (p. 255). Because these widely held beliefs are often fueled by a lack of information, we provided evidence-based answers to 11 concerns that managers express about employing people with disabilities. Our analysis, based on empirical evidence, supports inclusive employment practices that go beyond mere legal compliance. Indeed, the empirical literature reviewed in this paper reveals that across the employment cycle, workers with disabilities should not be cause for concern for employers. Rather, employers would be wise to make use of this underutilized labor pool, given the return on investments afforded by inclusive organizational practices.

In this paper, we have provided an overview of the concerns expressed by managers about hiring workers with disabilities, as well as used the current literature in management, human resources, industrial/organizational psychology, rehabilitation sciences, and public health to examine the validity of these concerns. In future work, the concerns along the employment cycle could be mirrored by focusing on the employees’ perspective. For example, the concerns expressed by managers surrounding accommodations are, from the perspective of employees with disabilities, concerns of appropriate provision of support. Similarly, concerns about performance go hand-in-hand with provision of accurate and timely feedback from the employees’ point of view. Managers’ concerns of organizational integration can be experienced by employees as a disjunction between attitudes and behaviors.

To be sure, some of the concerns expressed by managers, such as those surrounding organizational integration, may be relevant to other groups who are stigmatized in the workplace. Others, such as the concerns surrounding accommodation costs, or safety behaviors, are not. We have kept our focus on disabilities, which has provided us with a greater opportunity for an in-depth analysis. Thus, in addition to evidence-based responses to managers’ concerns, we have also provided managers with practical recommendations and additional resources that they may find useful if they seek to support workers with disabilities throughout the employment cycle. Finally, we have provided suggestions for additional research that further addresses these concerns. Our intention was to provide a starting point for a consideration of the experiences of workers with disabilities; given the considerable potential of this segment of the workforce, we should endeavor to leverage their abilities.

## Footnotes

1 Readers interested in international perspectives on disabilities may be interested in the World Bank World Report on Disability (http://documents.worldbank.org/curated/en/665131468331271288/Main-report) as well as the Disability Rights Education and Defense Fund list of relevant laws around the world (https://dredf.org/legal-advocacy/international-disability-rights/international-laws/).

2 Given that managers underestimate the prevalence of workers with disabilities in applicant pools, it is not surprising that they also do so within their own organizations. For example, a recent survey comparing health benefits plan sponsors (organizations) and plan members (employees) found that employers underestimated how many of their employees lived with a chronic condition by almost 50% (Sanofi Canada, 2016).

3 Customized employment typically involves applicants with severe disabilities. This approach requires “individualizing the employment relationship between employees and employers in ways that meet the needs of both. […] Customized employment assumes the provision of reasonable accommodations and supports necessary for the individual to perform the functions of a job that is individually negotiated and developed” (Federal Register, 2002, p. 43149–54).

4 While we discuss accommodations as the last concern in the selection portion of the employment cycle, it is important to point out that accommodation can take place throughout the employment cycle, from recruitment (e.g., providing an accessible application portal) to performance management (e.g., providing accommodation to address a possible performance issue). The relevance of accommodations to the entire employment cycle is reflected in our figure.
